# Treatment outcome and efficacy of an aligner technique – regarding incisor torque, premolar derotation and molar distalization

**DOI:** 10.1186/1472-6831-14-68

**Published:** 2014-06-11

**Authors:** Mareike Simon, Ludger Keilig, Jörg Schwarze, Britta A Jung, Christoph Bourauel

**Affiliations:** 1 Oral Technology Medical Faculty, Dental School, University of Bonn, Welschnonnenstr, 17 53111 Bonn, Germany; 2 Department of Orthodontics, University Medical Center, Hugstetter. Str. 55, 79106 Freiburg, Germany; 3 Private Practice, Richard-Wagner-Str. 9-11, 50674 Köln, Germany

## Abstract

**Background:**

The aim of this study was to investigate the efficacy of orthodontic treatment using the Invisalign® system. Particularly, we analyzed the influence of auxiliaries (Attachment/Power Ridge) as well as the staging (movement per aligner) on treatment efficacy.

**Methods:**

We reviewed the tooth movements of 30 consecutive patients who required orthodontic treatment with Invisalign®. In all patients, one of the following tooth movements was performed: (1) Incisor Torque >10°, (2) Premolar derotation >10° (3) Molar distalization >1.5 mm. The groups (1)-(3) were subdivided: in the first subgroup (a) the movements were supported with the use of an attachment, while in the subgroup (b) no auxiliaries were used (except incisor torque, in which Power Ridges were used). All tooth movements were performed in a split-mouth design. To analyze the clinical efficacy, pre-treatment and final plaster cast models were laser-scanned and the achieved tooth movement was determined by way of a surface/surface matching algorithm. The results were compared with the amount of tooth movement predicted by ClinCheck®.

**Results:**

The overall mean efficacy was 59% (SD = 0.2). The mean accuracy for upper incisor torque was 42% (SD = 0.2). Premolar derotation showed the lowest accuracy with approximately 40% (SD = 0.3). Distalization of an upper molar was the most effective movement, with efficacy approximately 87% (SD = 0.2).

**Conclusion:**

Incisor torque, premolar derotation and molar distalization can be performed using Invisalign® aligners. The staging (movement/aligner) and the total amount of planned movement have an significant impact on treatment efficacy.

## Background

In 1999, the Invisalign® system was introduced to the orthodontic market as a system of treating mild malocclusions, such as minor crowding and space closure [1]. In the following years, the system developed: different attachment designs and auxiliaries such as Precision Cuts and Power Ridges were designed to enable additional treatment of difficult malocclusions. According to the manufacturer, Invisalign® can effectively perform major tooth movements, such as bicuspid derotation up to 50 degrees and root movements of upper central incisors up to 4 mm [2]. In reference to the literature, however, there is no consensus about the exact indications of this system’s treatment [3]. This may be because little is known about orthodontic therapy with removable thermoplastic appliances (RTAs). Prior publications on Invisalign® mainly cover technical aspects, materials studies and case reports [4,5]. Only a few studies have concentrated on the efficacy of the treatment: Kravitz et al. [6] evaluated the accuracy of anterior tooth movement using the Invisalign® system and reported a mean accuracy of 41%. The most effective movement was lingual constriction (47.1%), and the least accurate movement was extrusion (29.6%).

To date, no published data could be found concerning the efficacy of tooth movements such as molar distalization and incisor torque with removable thermoplastic appliances. Some authors doubt whether bodily movements or torque can be accomplished at all by RTA and therefore recommend using RTA only in cases where tipping movements are needed [7].

Consequently, the purpose of this clinical and experimental study was to investigate the treatment efficacy of Invisalign® aligners for the following three predefined tooth movements: incisor torque >10°, premolar derotation >10°, molar distalization >1.5 mm.

For this purpose, the amount of tooth movement predicted by ClinCheck® (=software developed by Align Technology in order to provide the doctor a virtual 3-D simulation of the planned orthodontic treatment based on the patients beginning situation and the doctor’s predescribed treatment plan) was compared with the amount achieved after treatment. Furthermore, the influence of auxiliaries (attachments/Power Ridge) as well as the staging (movement/aligner) and the patient’s compliance on the treatment were evaluated.

## Methods

### Study design and patients

Models of 30 patients were retrospectively assessed in the period between 2011 and 2012. The Invisalign system is a worldwide well known and accepted orthodontic appliance, and due to the retrospective character of the study, the local ethical committee of the University of Bonn granted us exempt status for our retrospective study.

Inclusion criteria were healthy patients, treated with Invisalign® and one of the three following tooth movements were required:

1) upper medial incisor torque >10°,

2) premolar derotation >10°,

3) molar distalization of an upper molar >1.5 mm.

Exclusion criteria were patients with systemic disease, syndromes and cleft lip and palate. All patients’ malocclusions were exclusively treated with Invisalign® aligners in a private orthodontic practice in Cologne, Germany. The influence of auxiliaries, such as attachments (temporarily bonded composite buttons) and Power Ridges (pressure lines close to the gingival margin), on the above-mentioned tooth movements was investigated:

For carrying out upper incisor torque (group 1), (a) a ‘horizontal ellipsoid attachment’ or (b) power ridges were used according to the manufacturer’s information. In group 2 (premolar derotation) (a) optimized rotation attachment’ or (b) no auxiliary and in group 3 with a (a) ‘horizontal bevelled gingival attachment’ or (b) no auxiliary was used. In all, 60 tooth movements (20 in each main group, 10 in each subgroup) were determined using a split-mouth design. Furthermore, the tooth movement was performed in isolation in the ClinCheck® thus it could be analyzed exclusively.

### Attachments and staging

The attachments were engineered by Align Technology to achieve predictable tooth movements and placed according to the Align technology attachment protocol (horizontal ellipsoid attachment, horizontal gingival bevelled attachment) [8] or automatically placed by the software (optimized rotation attachment). Regarding the treatment protocol of Align technology, velocities up to 2 degrees/aligner for rotation, up to 1 degree/aligner for incisor torque and up to 0.25 mm/aligner for distalization are possible. To investigate the influence of the staging on the treatment efficacy, the tooth movements were planned to be partly slower and partly faster (Table 1).

**Table 1:** Amount of planned movement

**Tooth movement**	Possible staging*	Maximal amount of movement**	Mean movement/aligner**	Mean staging/aligner**
Premolar Derotation w Att	2.0°	30.0°	17.8°	1.1°
Premolar Derotation w/o Att	2.0°	35.0°	20.1°	1.2°
Distalization w Att	0.25 mm	3.2 mm	2.7 mm	0.2 mm
Distalization w/o Att	0.25 mm	3.2 mm	2.6 mm	0.2 mm
Incisor Torque w Att	1.0°	28.0°	16.1°	1.2°
Incisor Torque w PR	1.0°	30.0°	15.9°	1.1°

w Att. = with Attachment; w/o Att. = without Attachment; w PR = with Power Ridge.

*Possible staging according to the treatment protocol of Align technology.

**Amount of planned movement according to ClinCheck/the technician.

### Scanning, segmentation and superimposition

**Figure 1:**
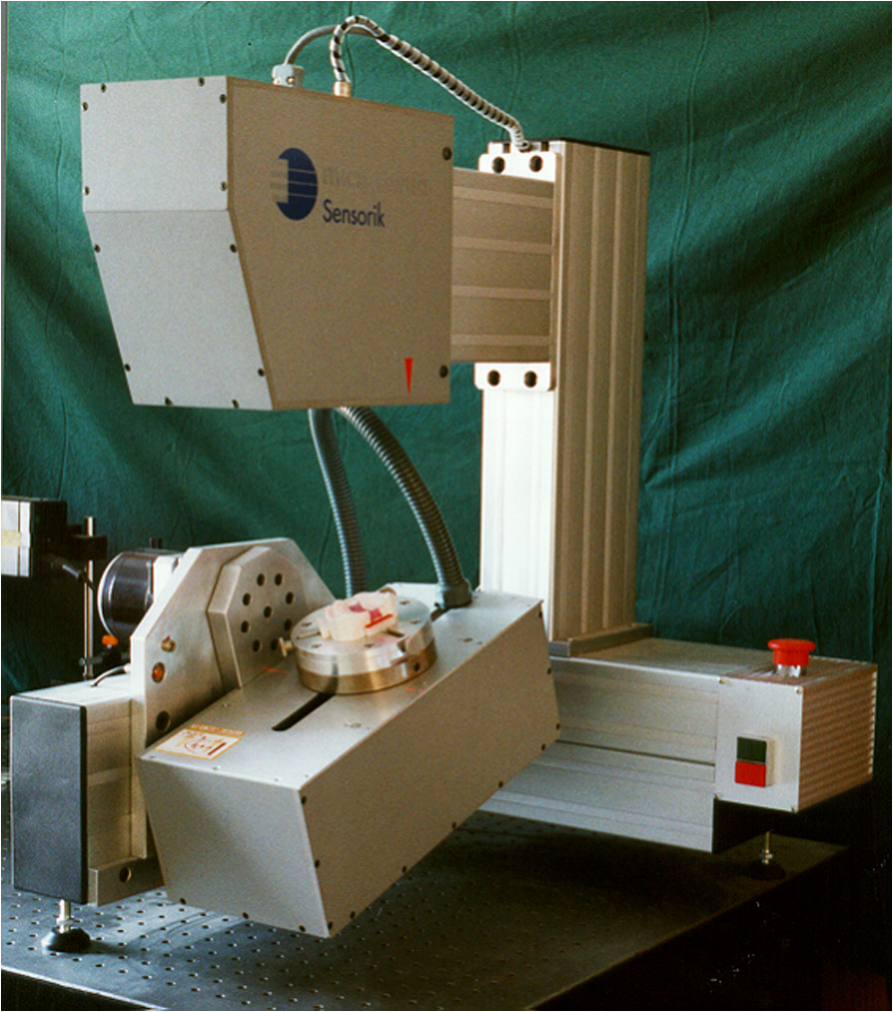
Laser scanner used in this study.

To document the clinical outcome, alginate impressions (Tetrachrom Alginat, Kaniedenta GmbH & Co. KG, Herford, Germany) of the intraoral conditions prior to the start of the movement _(T1)_ and immediately after finishing this treatment phase _(T2)_ were taken. The produced plaster cast models (Snow White Plaster, Kerr GmbH, Karlsruhe, Germany) were digitalized using a laser scanner (Micromeasure 70®, Microdenta Sensorik, Linden, Germany). Fixed on a motor-driven positioning table, the plaster casts were scanned on the basis of laser triangulation from four different predefined angles to cover all relevant areas and to prevent shadowing effects due to undercuts (Figure 1). The measuring points were registered by a charge-couple device (CCD) camera with an accuracy of approximately 20 μm according to the manufacturer’s data [9]. Thereafter, the individual scans were matched and merged into a single cloud of points by the computer to gain one single 3-D data set.

The ClinCheck® data at the time _T2_ (after finishing the investigated treatment phase) represented the virtual treatment goal. It was provided by AlignTechnology as a virtual 3-D model using the ClinCheck® database _(Clin T2)_.

**Figure 2:**
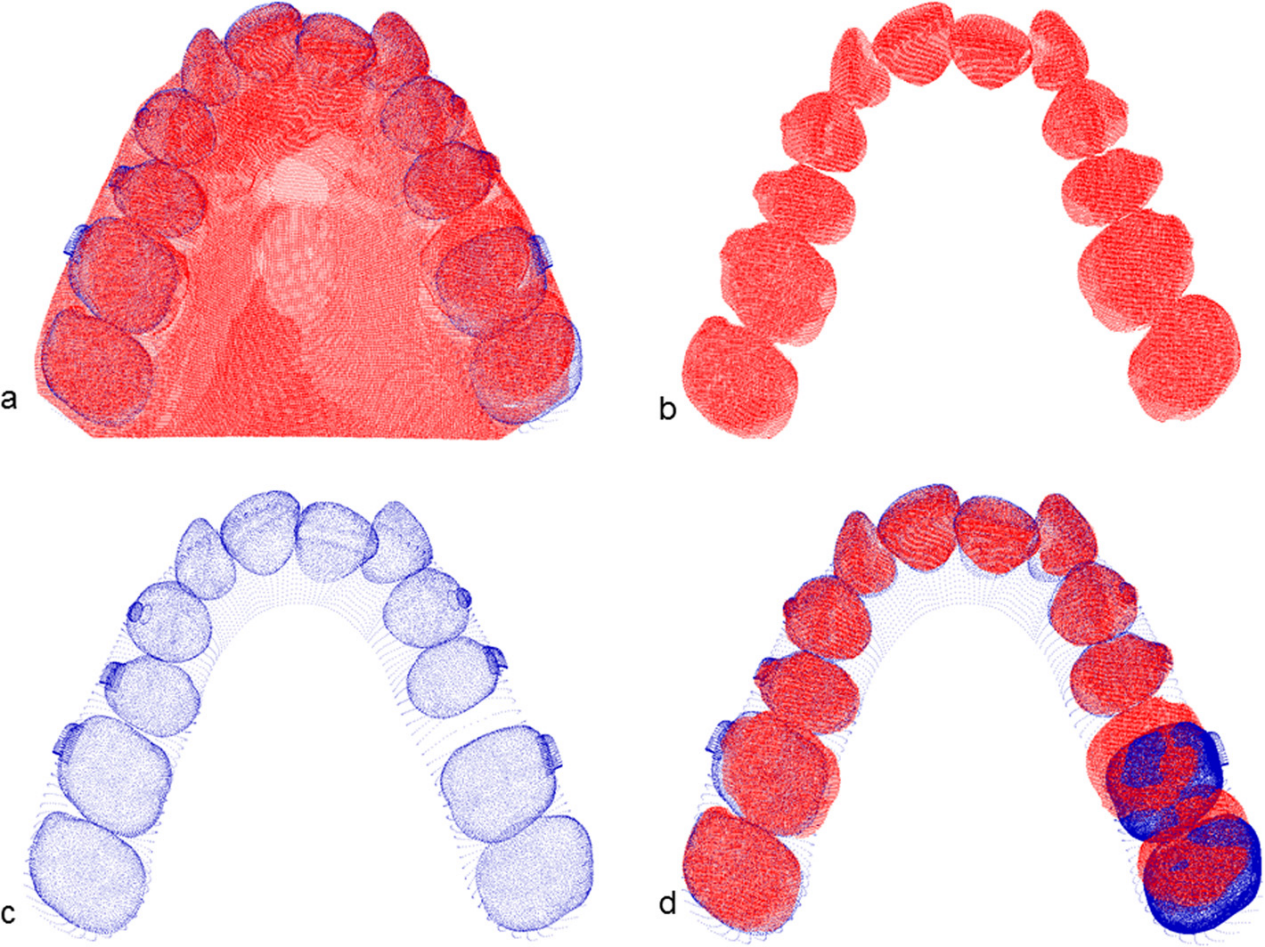
**Superimposition of the scans. a)** The plaster cast models of the beginning conditions are digitised using a laser scanner. **b)** The models are segmented into single teeth. **c)** The predicted tooth movement in the ClinCheck (extracted from the dataset from Align Technology). **d)** Superimposition of the plaster cast of the beginning conditions with the ending conditions in the ClinCheck to determine the predicted tooth movement.

The point of cloud of the pre-treatment _(T1)_, of the final plaster cast model _(T2)_ as well as the virtual 3-D ClinCheck® model _(Clin T2)_ were fed into the software Surfacer 10.0 (Imageware/Siemens PLM Software, Plano, Texas, USA). In the next step, each point of the cloud of _(T1)_, _(T2)_ and _(Clin T2)_ were segmented into the individual teeth (Figure 2). The cloud points of the untreated teeth of the initial situation _(T1)_ defined a global coordinate system for each patient and were used as a corresponding structure to merge the cloud points of the initial and final conditions. One after another, the clinically moved teeth of the final conditions _(T2 and Clin T2)_ were superimposed with the initial situation _(T1)_ using a surface/surface matching algorithm (Figure 2). In doing so the predicted movement by the ClinCheck® _(Clin T2 – T1)_ as well as the clinical achieved tooth movement _(T2 – T1)_ was determined exactly by the translational (Tx,Ty,Tz) and rotational (Rx, Ry, Rz) new coordinate components relative to the initial position [10].

To evaluate treatment efficacy, the difference between the parameters of the clinically achieved tooth movement _(T2 – T1)_ with the expected amount of tooth movement predicted by ClinCheck®_(Clin T2-T1)_ was calculated.

### Coordinate system

**Figure 3:**
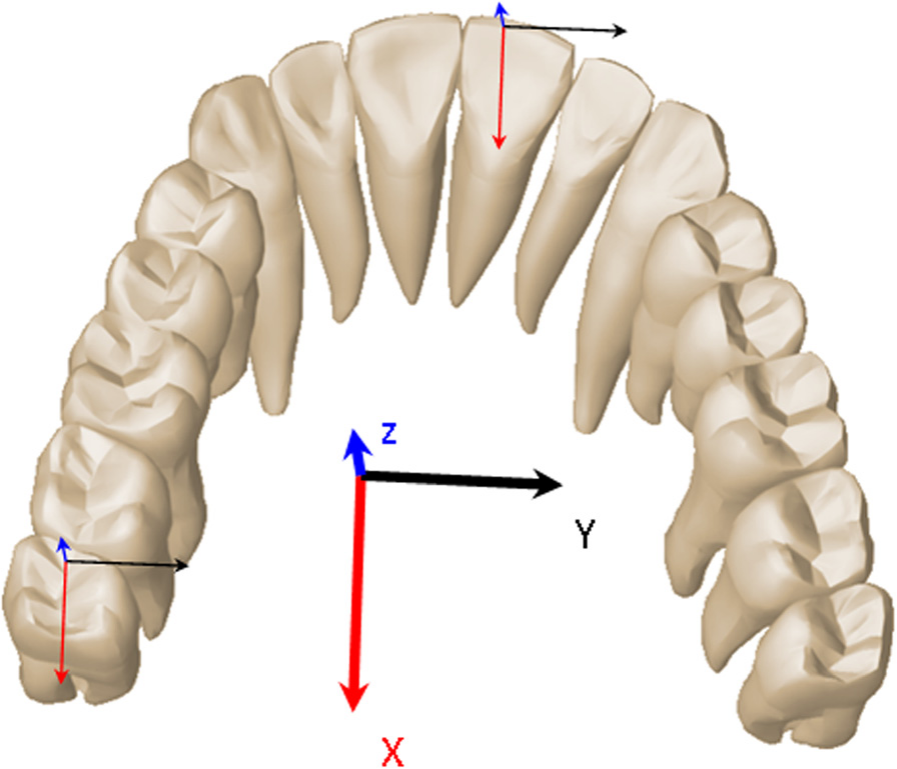
**Definition of the coordinate system used in this work.** The distalization is described as a translation on the x-axis, incisor torque as a rotation around the y-axis, and premolar derotation is described as a rotation around the z-axis.

To describe the tooth movement in all three spatial dimensions and to compare the predicted tooth movement with the achieved tooth movement, a reference coordinate system was set up (Figure 3): In the right-handed coordinate system, the axes were defined so that the x- and y-axes described movements in the horizontal plane and the z-axis described movements along the vertical plane. Thus, the tooth movement could be described by three translations (T_x,y,z_) and three rotations (R_x,y,z_) around the axes of this coordinate system. For the investigated tooth movements, that mean that an upper incisor torque was a rotation around the y-axis, premolar derotation was a rotation around the z-axis and molar distalization was a translation on the x-axis.

### Statistical analysis

The statistical evaluation included the analysis of the measured values as well as a minimum, a maximum, means and standard deviation of the mean. As the results were normally distributed according to the Shapiro-Wilk test, student’s paired t-test was used to analyze statistical difference between T2 (clinical achieved tooth movement) and ClinT2 (predicted tooth movement) values in each group (1–3: incisor torque, premolar derotation, molar distalization) for each subgroup (a: using an attachment, b: no attachment/using Power Ridge). A value of p ≤ 0.05 was considered statistically significant. The statistical evaluation was undertaken with the Statistical Package for Social Sciences, version 20.0 (SPSS Inc., Chicago, Illinois, USA).

## Results

### Clinical outcome

Of 30 patients (n = 11 male, n = 19 female; aged between 13 and 72 years, mean age 32.9 years, SD = 16.3), a total of 60 tooth movements were investigated (20 movements in each main group (1–3), 10 in each subgroup (a-b)).

However, 4 patients (13.3%) dropped out because:

– one patient moved away

– one patient discontinued orthodontic therapy

– impressions could not be taken from two patients directly after the investigated treatment phase (T2), since they did not re-attend at this point.

Therefore, the total amount of analyzed tooth movements revealed forty-nine:

– 14 in the incisor torque group (1), 7 in each group (a) and in group (b)

– 20 in the premolar derotation group (2), 10 in each group (a) and in group (b)

– 15 tooth movements in the distalization group (3), 7 in group (a) with attachment, 8 in group (b) without the support of an auxiliary.

Altogether, patients’ compliance was quite positive, with the exception of two patients who reported wearing their aligners for only 8 h per day, all patients followed the alignment technology treatment protocol of wearing their aligners the prescribed time of 22 h per day.

### Measurement outcome

**Figure 4:**
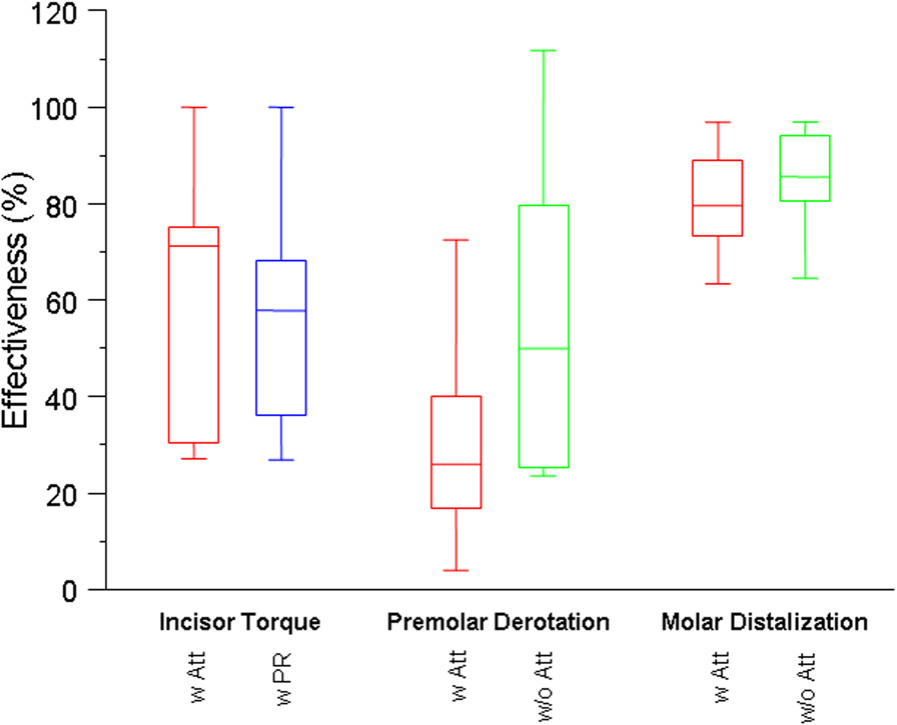
**Box– whisker plots showing the treatment efficacy of incisor torque**, **premolar derotation and molar distalization.** w. Att = with Attachment, w/o Att = without Attachment, w. PR. = With Power Ridge.

Figure 4 illustrates the treatment efficacy of the different tooth movements. The overall efficacy for all tooth movements amounted to 59.3% (SD = 0.2). The highest accuracy was achieved in the group of molar distalization, while the lowest accuracy was in the group of premolar derotation (Table 2). In the group of upper incisor torque (1) as well as in the group of premolar derotation (2) there was a statistical significant difference between the planned movement in the ClinCheck® and the clinical achieved movement (Table 2).

**Table 2:** Accuracy of tooth movements

**Movement**	**Mean accuracy (%)**	**Highest accuracy (%)**	**Lowest accuracy (%)**	**Standard deviation**	**p-value**
Premolar Derotation w Att	37.5	80.6	- 2.9	0.3	0.00
Premolar Derotation w/o Att	42.4	79.8	23.6	0.3	0.02
Distalization w Att	88.4	108.7	56.4	0.2	0.38
Distalization w/o Att	86.9	104.2	61.0	0.2	0.46
Incisor Torque w Att	49.1	71.6	29.9	0.2	0.00
Incisor Torque w PR	51.5	75.1	27.4	0.2	0.00

w Att. = with Attachment; w/o Att. = without Attachment; w PR = with Power Ridge.

p-value = clinically achieved tooth movement (T2) – tooth movement predicted by ClinCheck (Clin T2), p < 0.05.

Group 1: upper central incisor torque >10°:

No substantial differences were observed if the upper central incisor torque was supported with a horizontal ellipsoid attachment or with a Power Ridge. Measurements of incisor torque with Power Ridges (b) achieved a mean accuracy of 51.5% (SD = 0.2). The highest accuracy in this group was 75.1%, while the lowest accuracy amounted to 27.4%. In the group supported by an attachment (a), the mean accuracy amounted to 49.1% (SD = 0.2), the highest accuracy was 71.6%, and the lowest accuracy was 29.9%.

Group 2: premolar derotation >10°.

Also in the group of the premolar derotation no statistically significant difference could be found with regard to them being conducted in conjunction with an attachment. The mean accuracy achieved using an attachment (a) was 37.5% (SD = 0.3). The highest accuracy in this group amounted to 80.6%, and the lowest accuracy was -2.9%. This patient had poor compliance and claimed to have worn the aligner only 8 h per day. In the group of premolar derotation without the support of an attachment (b), the mean accuracy was 42.4% (SD = 0.3). The highest accuracy amounted to 79.8%, while the lowest accuracy was 23.6%. The efficacy of premolar derotation was further evaluated according to the amount of tooth movement as well as the amount of staging planned in the ClinCheck®: The results show that the accuracy was significantly reduced for predicted rotations greater than 15° as well as for rotations with a planned staging > 1.5°/aligner (Tables 3 and 4).

**Table 3:** Accuracy of premolar derotation according to the amount of planned tooth movement

**Planned movement**	**Mean accuracy (%)**	**Highest accuracy (%)**	**Lowest accuracy (%)**	**Mean standard deviation**
Premolar Derotation < 15°	43.3	72.5	16.8	0.24
Premolar Derotation > 15°	23.6	76.9	-2.9	0.15

**Table 4:** **Accuracy of premolar derotation according to the staging** (**movement**/**aligner**)

**Movement**	**Planned staging/aligner**	**Mean accuracy (%)**	**Mean standard deviation**
Premolar Derotation	< 1.5°	41.8	0.3
Premolar Derotation	> 1.5°	23.2	0.2

Group 3: molar distalization of an upper molar >1.5 mm.

The distalization of upper molars was the most effective movement, irrespective of the use of an attachment. The mean accuracy of molar distalization supported with an attachment (a) was 88.4% (SD = 0.2). The highest accuracy achieved was 108.7%, while the lowest accuracy was 56.4%. Without the support of an attachment (b), the mean accuracy for upper molar distalization amounted to 86.9% (SD = 0.16). The highest accuracy in this group was 104.2%, while the lowest accuracy was 61.0%.

## Discussion

It was the aim of our investigation to evaluate the treatment efficacy of three predefined tooth movements (translation, rotation and incisor torque) with aligners using the Invisalign® system, with respect to the influence of attachments/Power Ridges, the staging and the patients’ compliance.

In our study, the overall efficacy amounted to 59.3%. It should be noted, however, that the total efficacy in our study was composed of the efficacy of the three investigated movements: premolar derotation, molar distalization and incisor torque. Thus, it does not reflect the efficacy of complete orthodontic treatment.

Some authors doubt that bodily movements (especially incisor torque) can be accomplished using removable plastic appliances [7]. To generate the needed force systems, Invisalign® provides the use of an attachment or Power Ridge. As the results of our study indicate, both are practicable; nevertheless, a loss of torque up to 50% must be considered. However, it must be noted that the efficacy of fixed orthodontic appliances does not reach 100% either: Conventional orthodontic brackets and wires do not completely fill the bracket slots, so that the wire is able to twist, leading to a loss of moment, known as the so-called “torque play”. Moreover, the size and quality of the wire, the wire edge bevelling, the bracket material (polycarbonate brackets vs. metal and ceramic brackets) and bracket design, the interbracket distance, the vertical positioning of the bracket as well as the mode of ligation all influence the torque movement of conventional fixed appliances [11]. Due to this large amount of variation, it is almost impossible to calculate how much loss of torque expression exists with the use of a fixed appliance.

As described in the literature, one of the most difficult movements to perform with an aligner is the derotation of a cylindric tooth, as thermoplastic appliances tend to lose anchorage and slip off due to the presence of few undercuts and a round tooth shape [12,13]. This is reflected by the published results for premolar and canine derotation, which range between 29.1% to 49.7% [6,14-16]. In our study, the mean accuracy for premolar derotation (group 2) was 42.4% without and 37.5% with the support of an attachment. The lower efficacy in the group supported with an attachment was mainly due to poor patient compliance, which significantly reduced the treatment efficacy. It seems that if the aligner fitting is reduced but there is no attachment on the tooth’s surface, the rotational force transfer just decreases, whereas with an attachment, counter-moments can occur, leading to tooth movement in the opposite direction. If one was to exclude the patient’s poor compliance, a mean accuracy of 47.3% would be achieved. Overall, the amount of derotation influenced the accuracy significantly: If rotations greater than 15° were attempted, the mean accuracy of premolar derotation decreased by 46%, from 43.3% to 23.6%. These results were in accordance with those of Kravitz et al., who reported a significant reduction of up to 52.5% in the accuracy of canine derotation for rotations greater than 15° [6]. In addition to the amount of derotation, the staging (amount of derotation/aligner) also has a considerable impact on the treatment efficacy: for premolar derotations with a staging <1.5°/aligner, the total efficacy was 41.8% (SD = 0.3), whereas with a staging >1.5°/aligner, the accuracy decreased to 23.2% (SD = 0.2).

Among clinicians, one very important aspect is if and to what extent anterior-posterior movements can be performed using RTAs because this significantly increases the indications and allows for usage in even more complex malocclusions. Some authors reported a low accuracy of Invisalign® in correcting large anterior-posterior discrepancies [17]. To date, no scientific study has evaluated the exact efficacy of molar distalization using RTA. In our study, the molar distalization revealed the highest accuracy, approximately 87%. None of the patients used class II elastics during treatment. However, it should be noted that we measured the accuracy of distalization using a maximal amount of desmodontal anchorage: no anterior teeth were moved during the distalization of single molars. Furthermore, the anchorage lost in the posterior region during the retrusion of anterior teeth was not considered because the impressions were taken directly after the distalization of the second/first molar (T_2_). It remains to be investigated what impact simultaneous distalization of anterior teeth has on the overall efficacy of molar distalization, if the use of interarch elastic enhances distalization, and what amount of anchorage lost in the posterior region occurs during the retrusion of anterior teeth.

This study exhibited some limitations:

Because the data from the final tooth position in the ClinCheck® did not show the palatal surface, we used the untreated teeth as reference points for superimposition. Although only one tooth per hemiarch was moved, leaving enough teeth as a reference structure, relative movements of the reference teeth could not be excluded due to periodontal anchorage.

Furthermore, the aligner material we used in our study was the so-called the Exceed30 (EX30), the original aligner material from Align Technology. From the first quarter of 2013, a new aligner material called SmartTrack™ (LD30) was introduced to the orthodontic market by Align Technology. To what extent the new aligner material influences the treatment efficacy needs to be investigated.

Our evaluation focuses on the treatment efficacy of the three tooth movements during a certain set of aligners (on average 18) because during regular orthodontic treatment, the amount of aligners used to treat patients’ malocclusion is greater. In turn, the overall efficacy may be greater as the tooth movements are performed more slowly throughout the entire treatment time.

Another methodological deficit of this study was the low number of study participants, recruited from one single orthodontic practice. The treatment outcome using the Invisalign® appliance is strongly influenced by the experiences of the clinicians, so that the study results are not generally valid. To provide more accurate results on the treatment efficacy, a follow-up study with a larger sample size from several orthodontists would be useful.

Finally it must be said that we only investigated the efficacy of orthodontic treatment using the Invisalign® system with regards to the influence of auxiliaries (Attachment/Power Ridge), the staging (movement per aligner), as well as patient’s compliance. No comparison was made between the Invisalign® system and other orthodontic systems such as conventional fixed appliances, lingual appliances or other removable thermoplastic appliance systems. Further studies should compare treatment efficacy between different orthodontic treatment systems to find out which system is most appropriate for different dental malocclusion.

## Conclusions

This study showed that bodily tooth movements such as molar distalization, incisor torque, as well as premolar derotation can be accomplished using the Invisalign® system. Especially the efficacy of premolar derotation significantly depends on the velocity as well as the total amount of planed tooth movement. Upper incisor torque and pure premolar derotation are challenging movements using removable thermoplastic appliances - users should take into account that overcorrections or case refinements may be needed, since in these cases the ClinCheck® simulation could predict more movement than what may result clinically.

## Competing interests

The authors declare that they have no competing interests.

## Authors’ contributions

CB is the designer, supervisor and conductor of this project. MS participated in the project in all its phases (design, implementation, evaluation). LK supervised the experimental set up and the statistics. JS offered the patients’ models, BAJ is co-investigator and reviewed the manuscript. All authors read and approved the final manuscript.

## Pre-publication history

The pre-publication history for this paper can be accessed here:

http://www.biomedcentral.com/1472-6831/14/68/prepub
